# Improving diet, physical activity and other lifestyle behaviours using computer-tailored advice in general practice: a randomised controlled trial

**DOI:** 10.1186/1479-5868-9-108

**Published:** 2012-09-11

**Authors:** Sanjoti Parekh, Corneel Vandelanotte, David King, Frances M Boyle

**Affiliations:** 1 School of Population Health & Healthy Communities Research Centre, The University of Queensland Herston, Queensland 4006, Australia; 2 Institute for Health and Social Science Research, Central Queensland University, Rockhampton, Queensland Australia; 3 School of Medicine, The University of Queensland, Herston, Queensland 4006, Australia; 4 The University of Queensland, School of Population Health, Qld, 4006, Australia

**Keywords:** Health promotion, General practitioners, Intervention studies, Primary prevention, Diet, Physical activity, Health behaviours.

## Abstract

**Background:**

The adoption and maintenance of healthy behaviours is essential in the primary prevention of chronic non-communicable diseases. This study evaluated the effectiveness of a minimal intervention on multiple lifestyle factors such as diet, physical activity, smoking and alcohol, delivered through general practice, using computer-tailored feedback.

**Methods:**

Adult patients visiting 21 general practitioners in Brisbane, Australia, were surveyed about ten health behaviours that are risk factors for chronic, non-communicable diseases. Those who completed the self-administered baseline questionnaire entered a randomised controlled trial, with the intervention group receiving computer-tailored printed advice, targeting those health behaviours for which respondents were not meeting current recommendations. The primary outcome was change in summary lifestyle score (Prudence Score) and individual health behaviours at three months. A repeated measures analysis compared change in these outcomes in intervention and control groups after adjusting for age and education.

**Results:**

2306 patients were randomised into the trial. 1711 (76%) returned the follow-up questionnaire at 3 months. The Prudence Score (10 items) in the intervention group at baseline was 5.88, improving to 6.25 at 3 months (improvement = 0.37), compared with 5.84 to 5.96 (improvement = 0.12) in the control group (F = 13.3, p = 0.01). The intervention group showed improvement in meeting recommendations for all individual health behaviours compared with the control group. However, these differences were significant only for fish intake *(OR 1.37, 95% CI 1.11-1.68)*, salt intake *(OR 1.19, 95% CI 1.05-1.38)*, and type of spread used *(OR 1.28, 95% CI 1.06-1.51).*

**Conclusion:**

A minimal intervention using computer-tailored feedback to address multiple lifestyle behaviours can facilitate change and improve unhealthy behaviours. Although individual behaviour changes were modest, when implemented on a large scale through general practice, this intervention appears to be an effective and practical tool for population-wide primary prevention.

**Trial Registration:**

The Australian New Zealand Clinical Trials Registry: ACTRN12611001213932

## Introduction

Non-communicable diseases (NCDs) are, to varying degrees, associated with a limited set of modifiable health risk behaviours [1-5]. These behaviours are highly prevalent in developed countries; for example, 61% of the Australian population consumes more alcohol than recommended by current guidelines, 50% and 86% respectively fails to eat sufficient fruit and vegetables [6] and 50% is physically inactive [7] . These risk behaviours are not only highly prevalent but also occur in clusters; for example 99% of smokers had at least one additional risk such as unhealthy diet, high body mass index (BMI) or insufficient physical activity [8]. Clustering of unhealthy behaviours suggests the need for development and evaluation of interventions that target multiple health behaviours to achieve population health gains [8-10]. There are studies illustrating the limitations of self-regulatory capacity and the operating of concepts such as decision fatigue, indicating that it might be difficult for an individual to make multiple behavioural changes simultaneously [11]. However, multiple-behaviour change interventions are likely to have a greater impact on public health than single-behaviour interventions [4,10,12-14]. Only a limited number of such interventions have been evaluated to date, but generally show promising results [15,16]. Furthermore, there is evidence to suggest that interventions targeting more than one behaviour can be effective even when implemented simultaneously across the different behaviours [17-21]. To benefit cost-effectiveness and reduce participant burden, simultaneous interventions are preferred over interventions that target multiple behaviours sequentially [22].

The general or family practice setting offers potential to facilitate multiple behaviour change on a large scale, as approximately 85% of the Australian population consult a general practitioner (GP) each year [6]. Moreover, a GP’s advice is well accepted by patients [23] and reasonably effective in stimulating changes for certain habitual behaviours [24,25]. However, GPs experience crowded agendas and health promotion is often overlooked when patients present for management of acute conditions. A recent Australian study showed that GPs usually assess smoking and alcohol patterns but only about one in four typically assess their patient’s dietary and physical activity habits [26]. For interventions to be workable in general practice they must be easily accommodated within established practice routines and shown to have positive patient outcomes. A focus on multiple risk behaviours simultaneously offers a time-efficient approach. Computer-tailored health promotion interventions in this setting have shown promising results [27].

Interventions focused on diet and lifestyle that provide feedback tailored to an individual’s needs have demonstrated better feasibility [28,29] and effectiveness [27,30-33] when compared with non-tailored messages. Tailored messages are more likely to be read, remembered, discussed with others and perceived as interesting due to the personal relevance of the advice [34,35]. Computer-tailored feedback can be generated by an automated expert system, making it feasible to provide large number of respondents with personally adapted feedback about their present health behaviours [36].

Our previous work indicates that key aspects of diet and lifestyle can be reliably assessed by self-reported surveys [37]. There is growing evidence that multiple health behaviours can be summarised as a composite score and that higher scores are associated with lower morbidity and mortality [38-40]. A summary health score or a lifestyle score can be employed as a simple tool to communicate about the number of behaviours for which recommendations are met hence indicating the scope of change needed. However, little research has been published evaluating the use of such lifestyle score in achieving multiple health behaviour change in the general practice setting. Therefore, the aim of this study was to assess the effectiveness of a minimal intervention based around computer-tailored feedback derived from a summary health behaviour score of ten behaviours, within a representative general practice setting. It is hypothesised that the participants in the intervention group will significantly improve their health behaviours and increase their health score compared to the control group.

## Methods

### Study design and participants

Invitations were sent to 30 GPs in Brisbane, Australia, of whom 21 agreed to participate. The practice manager generated an initial list of patients, aged between 18 and 70 years, who had visited participating GPs in the preceding six months. The GP checked this list and excluded patients with active cancer, receiving renal dialysis, recent cardiovascular event, dementia, any other terminal illness or recent bereavement (n = 38). All communication with the patients originated from the research team, but used the treating doctor’s letterhead and electronic signature. Eligible patients received a postal invitation to participate, a study questionnaire and reply-paid envelope. Non-respondents were sent up to two reminder letters and a new copy of the questionnaire at three week intervals. Subjects who failed to respond at this stage were excluded. Return of the questionnaire was regarded as consent to participate in the project. Baseline data were collected for all participants from July to August 2008. The study was approved by the Behavioural and Social Sciences Ethical Review committee of the University of Queensland, Australia.

Patients who responded at baseline were randomised using a permuted block randomisation procedure [41]. This 2X2 factorial design randomised participants into intervention or control group, and for early (3 month) or late (12 months) follow-up. Further detail on the overall study design has been published earlier [42]. For each GP the block length was varied between 4, 8 or 12 to accommodate four study groups. Thus they were randomised into four groups as follows: intervention with 3 + 12 months follow-up, intervention with 12 months follow-up only, control with 3 + 12 months follow-up or control with 12 months follow-up only. Participants who resided at the same address were allocated to the same group as the first respondent from that address. Participants were blind to intervention condition, which was presented as a series of surveys followed by feedback. This paper focuses exclusively on participants reassessed at three months in order to examine the short term impact of the initial intervention. A forthcoming paper will address the sustainability of that change at 12 months and the impact of receiving or not receiving follow-up at 3 months. Behaviour change outcomes were assessed between Oct-Nov 2008 by re-applying baseline survey measures.

### Baseline measures

The baseline questionnaire used to calculate the Prudence Score has previously been validated [37] and includes 26 questions related to ten health behaviours and nine questions collecting demographic information. Responses to items addressing smoking, physical activity(using short version of International Physical Activity Questionnaire [43]), intake of alcohol, meat, fish, fruit and vegetables, use of unsaturated fats as spreads, avoidance of added salt, type of milk consumed, and body mass index (BMI, based on self-reported height and weight) were dichotomised. Each behavioural item was assigned a score of ‘1’ if achieving or exceeding recommendations, or a score of ‘0’. Scores were based on guidelines promulgated by the National Health and Medical Research Council (NHMRC) and the National Heart Foundation of Australia (NHF) (see Table1). Individual health behaviour scores were summed to yield a combined lifestyle score, the Prudence Score, ranging between 0 and 10. The remaining items in the baseline questionnaire addressed other health behaviours such as tetanus immunization, sun protection behaviour, non-smoking policies in private homes, and participation in mammography and cervical cytology screening. These items did not contribute to the Prudence Score. 

**Table 1:** **Socio-demographic and health behaviour characteristics at baseline (n = 1683), (mean** ± **SD for continuous data and percentages for categorical data)**

**Characteristics**	**Intervention (n = 853)**	**Control (n = 830)**	**Chi2 or F( p-value)**
**Demographics**			
Mean Age	49.2 ± 13.5	48.1 ± 13.5	3.00 (0.08^a^ )
Gender (% women)	68.5%	70.2%	0.62 (0.42^b^)
Full or part time employment	63.5%	66.5%	1.67 (0.19^b^)
Married or living as married	71%	70%	3.51 (0.66^b^)
Education (%Tertiary)	59.2%	55.5%	2.35 (0.12^b^)
**Health Behaviours**			
Meat intake ≤ 4 serves per week	70.2%	68.7%	0.44 (0.50^b^)
Fish intake ≥ 2 serves per week	68.1%	71.2%	0.36 (0.17^b^)
Use of low or no fat milk	70.6%	71.4%	1.90 (0.69^b^)
Salt: No added salt	46.2%	44.7%	0.36 ( 0.54^b^)
Vegetables and fruit : 7 serves per day	14.2%	12.4%	1.10 (0.29^b^)
Use of spreads other than butter	69.1%	67.4%	0.56 (0.45^b^)
Physical activity ≥ 150 minutes per week	48.4%	48.7%	0.01( 0.89^b^)
Alcohol ≤2 standard drinks per day	70.9%	71.4%	0.05 (0.80^b^)
No Smoking	87.0%	88.4%	0.75 (0.38^b^)
Body weight between 18.5 to 24.99 kg/m2	26.4 ± 5.6	26.5 ± 5.6	0.35 (0.56^a^)
**Mean Prudence Score**			
Total (n = 1599)	5.88 ± 1.6	5.84 ± 1.7	0.19 (0.66^a^)
Men (n = 495)	5.60 ± 1.6	5.44 ± 1.7	1.00 (0.31^a^)
Women (n = 1104)	6.01 ± 1.6	6.02 ± 1.6	0.01 (0.96^a^)

a ANOVA tested continuous variables.

b Chi-squared tested categorical variables.

### Intervention

All participants in the intervention group received only information related to the ten health behaviours comprising the Prudence Score. The intervention material consisted of:

(a) *Personalised computer-tailored feedback*: A one page personalised, computer-tailored feedback letter, printed on the treating practitioner’s letterhead, summarised the participant’s health score and indicated behaviours for which they were and were not meeting guideline recommendations.. This letter encouraged the adoption of at least one behaviour not currently contributing to the individual’s Prudence Score. The decision as to which additional behaviour(s) to improve was the patient’s own.

(b) *Health Promotion Information Material*: One page health promotion material was distributed to participants only for behaviours for which they were not meeting national guidelines, as indicated by their individual Prudence Score. For example, participants who did not meet NHMRC guidelines for vegetable intake but did meet recommendations for fruit intake only received the information sheet related to daily vegetable intake. The contents of the one page information sheet included the current guidelines for each particular health behaviour, some tips that may make it easier to adhere to this guideline, information related to the health benefits when adhering to the guideline and links where participants could find more information about this health behaviour from credible sources (e.g. the National Heart Foundation of Australia, the World Health Organisation, the Cancer Council Australia).

A publication by Kinzie draws on the recommendations of health behavioural theorists, and provides a unified framework from which to apply these theories in the design of health education. The authors offer a unified set of instructional design strategies for health education interventions by using a modified Events of Instruction framework (adapted from Robert Gagne) [44]. The framework includes five important strategies: gain attention, present stimulus material, provide guidance, elicit performance and provide feedback. Further, the intervention also applied the ‘Elaboration Likelihood Model’ by Petty and Cacioppo (1986) [45], which postulates that people will provide more attention to health information when it is perceived as personally relevant.

Similar to the intervention group, the control group received an individualised letter and tailored information sheets about the five health protective behaviours not included in the Prudence Score (sun protection, updating tetanus vaccination, mammogram and Pap smear). Feedback to the control group on these other behaviours was provided in an attempt to reduce attrition and to ensure both groups were treated comparably for a better test of the intervention. Participants in both groups received their personalised feedback within 10 days of the project team receiving their completed baseline survey. The processing of this information (data entry, generating the print-based personalised feedback using the custom software available to the project team and placing it in a new envelope) took on average 5 minutes per participant.

### Statistical analysis

Power calculations for the main study were based on pilot study results [37] (mean Prudence Score 4.94, SD 1.7). To have a 95% chance of the proportion with a Prudence score of 6 or more increase from 39% to 45%, using two-sided α = 0.05, required a total of 6600 invitations to participate, accounting for 20% loss to follow up and response fraction of 60% achieved in the pilot study.

Baseline differences in groups were analysed using one-way ANOVA for testing continuous variables and Chi-squared test for categorical variables. The primary outcome was change in mean Prudence Score at three months; this was assessed using T-tests and repeated measure ANOVAs. This analysis was adjusted for gender and age. The secondary outcome was measuring change in the proportion of participants adhering to individual behavioural items. This was measured using General Estimating Equations Models, in order to obtain odds ratios that examined associations between changes in individual health behaviours in the intervention group over and above the control group. Number-needed-to-treat (NNT) is the number of participants that needed to receive an intervention to achieve change in one individual. NNT was calculated using difference between change in control events and change in the experimental group. Inverse of that difference provided the number needed to treat. Initial analysis excluded participants with missing data at 3 month follow-up. However, this per protocol assessment was complemented by an intention-to-treat analysis, in which we assumed that patients lost to follow-up had not changed their behaviour. Significance was set at *P* < 0.05 for all analyses.

## Results

**Figure 1:**
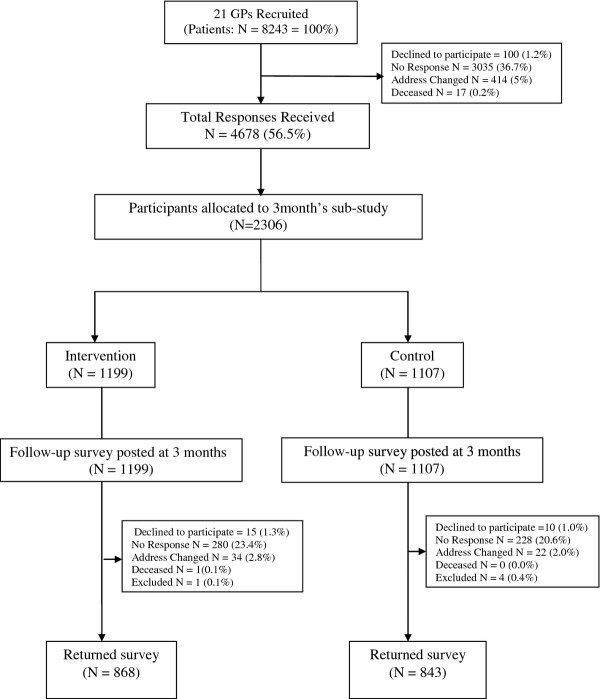
Flowchart of participant recruitment and randomisation.

Invitations and questionnaires were mailed to 8243 patients, after 38 were excluded by their treating GP. 4678 patients agreed to participate in the study by returning completed questionnaires, giving a baseline response of 56.5%, see Figure 1. Health behaviour changes reported are only for participants in the sub-study to be reassessed at 3 months (n = 2306). A total of 76% (n = 1711) participants responded at three months. Twenty nine participants had excessive missing data and were excluded; hence the final sample consisted of 1683 participants. Participants had a mean age of 48.6 years (SD = 13.5), mean BMI in the overweight range (26.4 kg/m2; SD = 5.4) and were predominantly female (69.7%). There were no significant differences between groups at baseline in gender, health behaviours (See Table 1), or mean Prudence score (5.88 versus 5.84, *P* = 0.65).

### Individual health behaviours

There was an overall improvement in health behaviours in both intervention and control groups at three months (Table 2). More improvement was noted in the intervention group compared to the control group, except for smoking prevalence (Table 2). However, significant changes between groups were observed for only three behaviours: increased fish intake (*OR 1.37, CI 1.11-1.64)*, reduced salt intake *(OR 1.19, CI 1.05-1.38)* and using spreads other than butter *(OR 1.28 CI 1.06-1.51)*. The odds ratios for change in intervention group compared to the control group, adjusted for age and gender, are included in Table 2. Participants in the intervention group were 40% more likely to increase their fish intake as compared to controls. Similarly, the intervention group were 20% and 30% respectively more likely to reduce salt intake and use of spreads other than butter. The number needed to treat (NNT) indicates, taking fish intake as an example, that for every 15 patients receiving the intervention one individual will adopt sufficient change to achieve guideline recommended fish intake. Apart from the minimal change seen in body weight and smoking, all other behaviours recorded NNT of between 15 and 58 (Table2).

**Table 2:** Net percentage change, odds of change and number needed to treat (NNT) for participants achieving guidelines recommendations (n = 1683)

**Health Behaviour**	**Group**	**Net change%**	**Odds Ratio for change**	**95% CI**	**NNT#**
Fish	Intervention	+7.06	1.37*	**1.11-1.64**	15
	Control	+0.84			
Spread	Intervention	+5.06	1.28*	**1.06-1.51**	21
	Control	+0.37			
Salt	Intervention	+5.43	1.19*	**1.05-1.38**	24
	Control	+1.23			
Veg and fruit	Intervention	+3.14	1.24	0.91-1.68	37
	Control	+0.49			
Meat	Intervention	+7.17	1.16	0.93-1.44	38
	Control	+4.48			
Milk	Intervention	+4.62	1.11	0.96-1.29	45
	Control	+1.80			
Alcohol	Intervention	+3.88	1.16	0.96-1.37	45
	Control	+1.12			
Physical activity	Intervention	+0.48	1.06	0.86-1.30	58
	Control	−1.34			
Body weight	Intervention	−0.12	0.96	0.85-1.09	161
	Control	−0.82			
Smoking	Intervention	+0.24	0.97	0.86-1.12	1000
	Control	+0.36			

*Significant Results (p < 0.05).

#NNT : Number needed to treat.

### Prudence score

The change in the Prudence Score from baseline to three months was significantly greater in the intervention group (5.88 to 6.25, difference + 0.37) when compared to the control group (5.84 to 5.96, diff = 0.12) (F = 13.3, p = 0.01). (Table 3) Similar changes at 3 months were observed for men (F = 4.6, *P* = 0.03) and women (F = 8.6, *P* = 0.003). The participants who were lost to follow-up at three months had significantly lower Prudence Score at baseline compared to continuing participants (5.35 versus 5.85, F = 2.8, p = 0.02). However, with an intention-to-treat analysis the mean Prudence Score still showed significant increase in the intervention group compared to the control group (6.02 versus 5.74, F = 11.58, P < 0.001).

**Table 3:** Effect of intervention on the Prudence Score at 3 months (n = 1599)

	**Intervention**	**Control**	***T*-test (p value)**^**a**^	**Repeated Measures ANOVA**^**b**^	**Repeated Measures ANOVA**^**c**^
	**Prudence Score (95% CI)**	**Prudence Score (95% CI)**			
**Total**					
Baseline	5.88 (5.77-5.99)	5.84 (5.73-5.96)	t = 0.44 (p = 0.66)	F = 13.3 P = 0.01	F = 11.58 p < 0.001
3 months	6.25 (6.13-6.36)	5.96 (5.84-6.08)	t = 3.47 ( p = 0.001)		
**Net Change**	**0.37**	**0.12**			
**Men**					
Baseline	5.60 (5.39-5.80)	5.45 (5.22-5.67)	t = 0.99 (p = 0.32)	F = 4.6 p = 0.03	F = 4.0 P = 0.04
3 months	5.92 (5.72-6.13)	5.55 (5.33-5.77)	t = 2.45 (p = 0.014)		
**Net Change**	**0.32**	**0.10**			
**Women**					
Baseline	6.01 (5.88-6.14)	6.01(5.88-6.16)	t = 0.04 (p = 0.96)	F = 8.6 p = 0.003	F = 7.6 P = 0.006
3 months	6.40 (6.26-6.54)	6.13(5.99-6.27)	t = 2.72 (p = 0.006)		
**Net Change**	**0.39**	**0.12**			

a *T*-test is used to assess the difference between intervention and control group at baseline or 3 months in the P-Score.

b ANOVA adjusted for gender and age.

c ANOVA adjusted for gender and age with intention-to-treat analysis.

When participants were categorised into 3 groups according to the baseline Prudence Scores (Table 4), the maximum improvement in Prudence Score was observed in the low baseline score group, an increase of 1.18 in the intervention group, but low scoring controls also improved their Prudence score by 0.82(net difference +0.36). Both groups of participants with high baseline scores reported a decline in Prudence scores at 3 months, but a significantly lesser reduction in the intervention group (net difference of +0.61) suggested that the intervention was possibly most effective in participants already following predominantly healthy behaviours.

**Table 4:** Effect of intervention on the P-Score (n = 1599) for high, medium and low scores at baseline

***Prudence Score Category#***	***Mean score at baseline (SE)***	***Mean score at 3 months (SE)***	***Net difference***	Repeated measures ANOVA*
***Low Scorer (0–4)***				
Intervention	3.40 (0.047)	4.58 (0.113)	1.18	F = 4.18 p = 0.04
Control	3.44 (0.053)	4.26 (0.109)	0.82	
***Medium Scorer (5–7)***				
Intervention	6.00 (0.030)	6.32 (0.059)	0.32	F = 6.82 p = 0.01
Control	5.92(0.031)	6.04(0.058)	0.12	
***High Scorer (8–10)***				
Intervention	8.33 (0.048)	8.04 (0.101)	−0.29	F = 4.97 p = 0.03
Control	8.33 (0.044)	7.33 (0.109)	−1.0	

*ANOVA adjusted for gender and age.

# Participants grouped into 3 score categories based on their baseline health scores.

## Discussion

The primary aim of this study was to assess the effectiveness of a minimal, computer-tailored intervention in a primary care setting using a summary health behaviour score. This intervention was effective in increasing the lifestyle score over a three month period, although a statistically significant positive change was observed in only three individual health behaviours. The implications of this study are supported by primary prevention studies that have demonstrated morbidity and mortality benefits from lifestyle behaviour change [40,46,47].

Study participants showed a significant increased adherence to guidelines for salt intake, fish intake and type of spread used. A similar increase in fish consumption was observed in an intervention study by Sacerdote et al. [48]. Other interventions targeting self-reported dietary intake in primary care populations have produced only small improvements, consistent with our results on fruit and vegetable intake [49]. The three behaviours that showed significant improvements are probably easier to adopt due to simple substitution, for example using margarine rather than butter. Behaviours such as increasing physical activity or smoking cessation probably require greater organisation and motivation. It is likely that the minimal intervention provided was insufficient to enhance organisational skills or able to reinforce motivation to change these complex behaviours. None the less , success in adopting easy behaviours might increase confidence and self-efficacy to facilitate subsequent attempts at more difficult to change behaviours [50].

The response fractions at baseline (57%) and three months (76%) are comparable to other studies undertaken in general practice settings [51]. The strategies used in this trial, such as repeat mailing to initial non-responders, GP endorsement and shorter questionnaires, have been shown to improve response to postal questionnaires in health care research [52]. GP endorsement of invitation letters and questionnaires may have improved the extent of behaviour change detected, due to the authority and esteem held by patients for their doctor.

Women in the study had significantly higher Prudence Scores than men at baseline and at three months. The change over time was also greater in women; though not significantly so. Similar gender differences in health have been observed in other studies [53,54]. This difference could be partly due to women accompanying their children for medical attention and their more regular attendance for contraception and screening tests. Women make up 60% of visits to Australian general practices [55] and are therefore more likely to receive greater exposure to health information. However, our finding that this intervention was equally effective in changing behaviours in both men and women might provide opportunities to close the gender gap. As men are traditionally harder to reach with health messages than women [56], the minimalist nature of our intervention might be better suited for them.

Limitations of our study include firstly the dichotomous scoring system for health behaviours, where sub-threshold change in behaviour remains undetected. Yet this would suggest that our results might have underestimated the real extent of behaviour change. However, the Prudence Score is designed for simplicity in order to provide easy to interpret feedback about health behaviours to large numbers of patients. Secondly, the ten component behaviours are equally weighted in their contribution to the Prudence Score, rather than being weighted according to their relative impacts on health. Dietary factors are over represented compared to exercise and smoking, which only contributed a single score each. A study employing an equally weighted lifestyle scoring system used all the same items as the Prudence Score ( except vegetable and fruit intake and type of spread ) to predict mortality in both healthy elderly men and older men with established vascular disease [39,57]. It was able to demonstrate a linear relationship between increasing lifestyle score and decreasing mortality rate with an absolute reduction in cumulative mortality of 0.62% per single additional healthy behaviour [57] . This suggests that the aggregate score is a meaningful summary of an individual’s effort to protect their health. However, assessing the direct impact of the Prudence score on morbidity and mortality is beyond the scope of this trial. Thirdly, the use of self-reported data was a potential weakness: however the assessment questionnaire was previously validated [37] and our study participants were representative of the wider Australian population [6,58]. Finally, as this project provided print-based feedback to participants the process of data collection and feedback provision was labour intensive. However, a fully automated implementation, applying waiting-room kiosks or tablets, can easily be developed based on this study. Further research is needed to determine the effectiveness of a fully automated version of this intervention.

Our study had several strengths: a randomised design with allocation concealment, measurement of individual as well as aggregate changes in health behaviours and a large sample size. Lifestyle interventions focused on prevention must be effective but also available and accessible to the public. The ten behaviours in the Prudence Score can be measured by an individual without the help of a health professional. Lifestyle changes arising from this intervention were achieved without face-to-face intervention or planned GP advice and are likely to be cost effective outside the research setting. A particular advantage of this intervention is its population coverage given very high levels of access to primary care in Australia [6].

## Conclusion

Finding feasible and innovative ways to use technology for improving health behaviours in large numbers of individuals is vital for the primary prevention of NCDs. Geoffrey Rose’s notion that population-wide strategies are likely to be more effective than those that focus on high-risk individuals [59] calls for research into such minimal, wide-reach interventions. This study contributes to this research agenda by extending the limited evidence currently available in the field of multiple health behaviour change intervention trials in the general practice setting. Although the individual behaviour changes resulting from this intervention were relatively modest, the Prudence Score, which can be implemented on a large scale and is easy to calculate, appears to be a useful tool for improving behaviours in primary prevention.

## Abbreviations

BMI: Body mass index; GP: General practitioner; NCD: Non-communicable disease; NHF: National heart foundation; NHMRC: National health and medical research council; NNT: Number needed to treat.

## Competing interests

The author(s) declare that they have no competing interests.

## Authors contributions

SP recruited the general practitioners and study participants, collected and analysed the data and drafted the manuscript. DK participated in recruiting general practitioners. CV also provided supervision in generating tailored feedback and health promotion materials. DK, FB and CV helped to draft the manuscript. SP, CV, DK, and FB read and approved the final manuscript.
